# Health Risk Assessment of Heavy Metals in Shallow Groundwater of Coal–Poultry Farming Districts

**DOI:** 10.3390/ijerph191912000

**Published:** 2022-09-22

**Authors:** Jiayu Chen, Herong Gui, Yan Guo, Jun Li

**Affiliations:** 1 School of Earth and Environment, Anhui University of Science and Technology, Huainan 232001, China; 2 National Engineering Research Center of Coal Mine Water Hazard Controlling, Suzhou University, Suzhou 234000, China; 3 School of Resources and Environmental Engineering, Hefei University of Technology, Hefei 232000, China

**Keywords:** heavy metals, source identification, health risk assessment, poultry farms sites, PMF model

## Abstract

This study aimed to assess the heavy metal (Mn, Ni, Cu, Zn, Sr, Cd, Pb, and Cr) pollution characteristics, sources, and human health risks in shallow groundwater in the impact zones of urban and rural semi-intensive poultry farms in Suzhou City. Ordinary kriging interpolation showed that poultry farming contributed substantially to the pollution of shallow groundwater by Mn, Zn, and Cu. Positive matrix factorization was applied to identify the sources of heavy metals, and the health risks were assessed based on the hazard index and carcinogenic risks of the various sources. Heavy metal enrichment was closely related to anthropogenic activities. In addition, four sources were identified: poultry manure (29.33%), natural source (27.94%), industrial activities (22.29%), and poultry wastewater (20.48%). The main exposure route of carcinogenic and non-carcinogenic risks to adults and children was oral ingestion. The non-carcinogenic risk of oral ingestion in children was higher than that in adults; the carcinogenic risk was higher in adults than in children. Poultry manure (42.0%) was considered the largest contributor to non-carcinogenic risk, followed by poultry wastewater (21%), industrial activities (20%), and natural sources (17%). Industrial activity (44%) was the primary contributor to carcinogenic risk, followed by poultry wastewater (25%), poultry manure (19%), and natural sources (12%).

## 1. Introduction

Shallow groundwater is the primary water source for people’s daily lives in most areas; approximately one-third of the world’s population uses groundwater as a source of drinking water [1]. Human activities, such as mining, industry, and poultry breeding, have aggravated the discharge of heavy metals into shallow groundwater, thereby affecting human health [2,3,4]. The intake of heavy metals can substantially affect human health, even at trace doses [5]. For example, excessive intake of Cr can cause lung cancer, kidney damage, and other diseases [6]. The accumulation of Cu, Mn, Zn, Cd, and Pb in the human body can cause kidney damage and cancer risk [7,8].

In recent years, with the rapid development of the breeding industry, the environmental problems brought by the livestock and poultry breeding industry have attracted widespread attention. In 2017, China’s total discharge of livestock and poultry manure was 1.64 × 10^9^ t (FW) [9]. Since livestock manure contains heavy metals and antibiotic resistance genes [10,11], the unreasonable discharge of livestock manure and wastewater poses a potential threat to human health. Agriculture, livestock, and poultry farming are the first and second largest sources of chemical oxygen demand (COD) and ammonium nitrogen (NH_4_^+^-N). The COD, total nitrogen, and total phosphorus released in the water environment from livestock and poultry farming accounted for 41.9%, 21.7%, and 37.9% of the overall wastewater discharge, respectively [12]. The manure load from semi-intensive or intensive poultry farms has exacerbated the heavy metal pollution of groundwater through rainwater and fertilization leaching, gravely deteriorating the quality of shallow groundwater [5]. The current research mostly focuses on heavy metal [1] and antibiotic [9,10] pollution in the feces or sewage of poultry farms or the impact of farming operations on surface water [13]; however, the heavy metal pollution of shallow groundwater in the affected areas and health risk assessment studies are still limited.

Ordinary kriging (OK) interpolation is a linear transformation space interpolation technique [14], which can be used to illustrate the spatial distribution of heavy metals, and it has been widely used in the research of groundwater contamination [14,15,16]. Positive matrix factorization (PMF) models are used to quantitatively determine pollution sources and their contribution [17,18]. PMF is a least squares-based factor analysis model that has been widely used in the past decades for air pollution [19,20], heavy metals and dissolved organic matter (DOM) in soil, and surface water [21,22,23,24,25] pollution research. The PMF model takes into account a single error estimate [26] that handles outliers and non-representative data (missing values and BDL below the detection limit). Although PMF is used to address air, soil, and surface water pollution, little information is available regarding its applicability in groundwater pollution assessment and source apportionment. This paper reveals the sources and distribution characteristics of eight heavy metals (Mn, Ni, Cu, Zn, Sr, Cd, Pb, and Cr) in shallow groundwater in areas affected by poultry farms, providing a reference for similar areas in other regions.

Suzhou City is located in the northern part of Anhui Province, and its main industry is poultry breeding in Zhuxianzhuang Town (eastern region). In this region, poultry farming is mostly semi-intensive and resource-based. The produced manure is concentrated in septic tanks for natural degradation, and the degraded waste is used as field fertilizer. The town has a population of approximately 100,000. Shallow groundwater is the main water source for drinking, industries, agriculture, and aquaculture. Therefore, the pollution of shallow groundwater affects the health risk of adults and children in the area.

The objective of this paper was to (1) identify the possible sources of heavy metals through GIS spatial distribution and PMF model, (2) assess the non-carcinogenic and carcinogenic risks of heavy metals to human health.

## 2. Materials and Methods

### 2.1. Site Description

**Figure 1:**
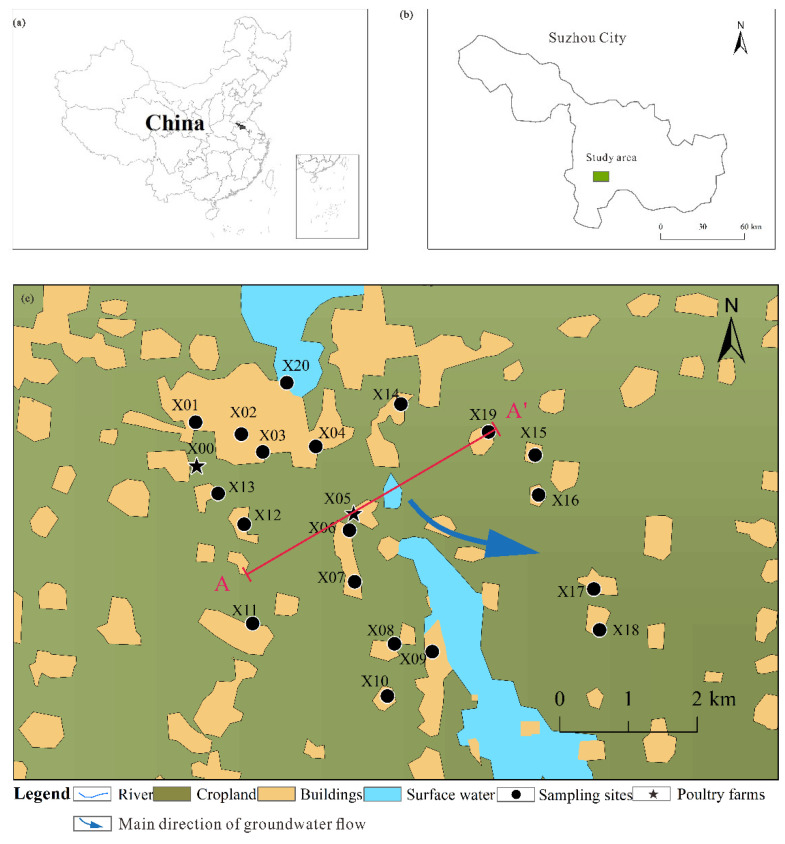
Location of study area and distribution of water sampling points. (**a**) China; (**b**) the location of study area; (**c**) the location of sampling points and land use.

**Figure 2:**
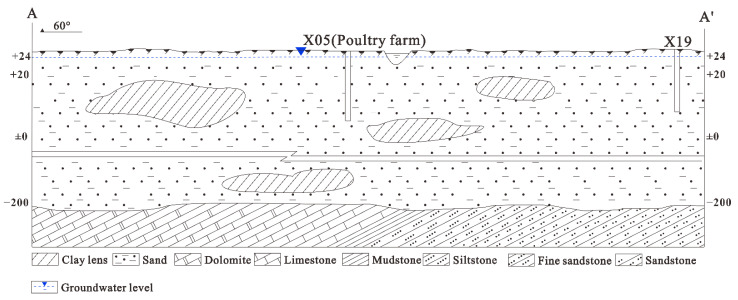
Simplified hydrogeological cross-section A−A′.

Suzhou has a population of 6.5 million and area of 9787 km^2^ (Figure 1b), and the average annual rainfall and evaporation in Zhuxianzhuang Town are 774–855 mm and 832.4 mm, respectively. Corn, soybean, and wheat are the main crops cultivated in the town. The thickness of the Cenozoic Quaternary loose layer in the study area is 200–300 m, divided into four aquifers. Shallow groundwater is the first of four aquifers (from top to bottom), with an average depth of 30 m, primarily composed of silt, fine sand, and clay (Figure 2). The shallow groundwater is mainly recharged by atmospheric precipitation or surface water via infiltration and excreted through infiltration or evaporation. Surface water recharges groundwater much more than groundwater recharges surface water [27]. Shallow groundwater had a permeability coefficient *k* = 0.86–12.25 m/d; seepage velocity *v* = 0.56 × 10^−4^–0.12 × 10^−2^ m/d; hydraulic gradient *i* = 1/10,000–0.65/10,000. Affected by the water blocking effect, the third and fourth aquifers are mainly supplied by interstitial recharge, with a poor water volume [28]. Due to similar lithological characteristics, shallow groundwater will be analyzed together with deep groundwater as an integral constituent of the groundwater circulation system.

The study area is located within latitudes 33°33′24″ N to 33°37′37″ N and longitudes 117°5′29″ E to 117°9′20″ E (Figure 1c), with a total area of approximately 48.77 km^2^. The study area houses dozens of large-scale poultry farms and several small family farms. The selected poultry farms operate on a semi-intensive scale and might directly discharge the sewage and wastewater that wash away manure to the farmland without treatment, inevitably polluting the shallow groundwater. Therefore, two semi-industrial poultry farming bases were selected as research sites in this study. More than 5000 chickens are produced in each farm annually. Of note, as the study area includes a coal industrial park and two semi-intensive poultry farms, mining activities and farming operations may affect the quality of shallow groundwater in the surrounding area, so the term “coal–poultry” was used to refer to anthropogenic activities related to the coal industry and poultry farming.

### 2.2. Collection and Analysis of Water Samples

In August 2020, water samples were collected from 21 monitoring wells (Figure 1c). Samples were sealed in 21 polyethylene sampling bottles (250 mL), pumped continuously for 5 min before sampling, and washed three times with fresh well water. Most specimens were sampled from loose Quaternary rock pore phreatic water, with the depth of most wells being less than 30 m. After the collection, the samples were sent to the laboratory of the National Engineering Research Center of Coal Mine Water Hazard Controlling in Suzhou and stored in a refrigerator at 4 °C. After suction filtration through a 0.45 μm filter membrane, the samples were collected in 50 mL sampling bottles and acidified to a pH of 1.5–2.0 with about 3 mL of 65% nitric acid. The concentrations of Mn, Ni, Cu, Zn, Sr, CD, Pb, and Cr were measured by inductively coupled plasma mass spectrometry (ICP–MS) within one week.

The correlation coefficient of the standard curve was above 0.999, and a standard sample (GSB 04-1767 2004) was used to fit the standard curve using the external standard method. Program blanks, blind repetitions, and standard reference materials were analyzed for quality assurance and quality control of the obtained data. The standard recovery rate of each element was between 80–120% and the error of the experimental analysis was controlled within 5%. The experiments were conducted in triplicate, and their average values were used. Moreover, a 10% parallel double sample was set. The limit of detection (LOD) for Mn, Ni, Cu, Zn, Sr, Cd, Pb, and Cr was 0.01349, 0.01393, 0.02304, 0.05357, 0.001, 0.01393, 0.00036, and 0.00665 µg/dm^3^, respectively. The limit of quantitation (LOQ) was 0.10, 0.12, 0.20, 0.26, 0.093, 0.01, 0.056, and 0.053 µg/dm^3^, respectively. The test results of all samples met the quality control requirements (qualified rate of 100%).

### 2.3. PMF

PMF was applied to quantitatively determine the pollution sources and contribution ratios of eight heavy metals, namely Mn, Ni, Cu, Zn, Sr, Cd, Pb, and Cr [17,18]. PMF is a factor analysis model based on the least square method. The sample data was decomposed into two matrices: factor contribution and factor distribution. The main advantage of this method is that it enabled the estimation of uncertainty and its association with sample data, in addition to the easy management of lost data. The simulation results of the PMF model were correlated with environmental variables to identify the main source corresponding to each factor [29]. PMF was calculated as follows:

(1) $$X_{ij}=\sum_{k=1}^{p}g_{ik}f_{kj}+e_{ij},$$

(2) $$Q=\sum_{i=1}^{n}\sum_{j=1}^{m}{\left\{\frac{X_{ij}-\sum_{k=1}^{p}g_{ik}f_{kj}}{u_{ij}}\right\}}^{2},$$

where *X_ij_* is the measurement matrix of the *j*th element in the *i*th sample, *g_ik_* is the contribution matrix of the *k*th source factor of the *i*th sample, *f_kj_* is the source profile of the *j^th^* element of the *k*th source factor, *e_ij_* is the residual value of the *j*th element in the *i*th sample, and *u_ij_* is the uncertainty of the *j*th heavy metal element in the *i*th sample.

The uncertainty was expressed as follows:

(3) $$u_{ij}=\frac{5}{6}\times MDL,\;\mathrm{when}\;c\;\leq MDL;$$

(4) $$u_{ij}=\sqrt{{\left(EF\times c\right)}^{2}+{\left(0.5\times MDL\right)}^{2}},\;\mathrm{when}\;c>MDL;$$

where *c* is the concentration of elements in the sample, *MDL* is the detection limit of the determination method, and *EF* is the corresponding precision.

### 2.4. Health Risk Assessment

According to the US Environmental Protection Agency (USEPA), the health risk assessment can be divided into non-carcinogenic and carcinogenic evaluation [17,18]. The exposure pathways associated with these risks include, among others, direct intake and skin contact. These two pathways and two groups of people (adults and children) were considered in this study. In this study, the assessment factors in evaluating the environmental risk of shallow groundwater included Ni, Cd, Pb, and Cr as carcinogens health risk assessment models [17,30]; However, chemical carcinogens also exert non-carcinogenic effects [17,30]. Therefore, Mn, Ni, Cu, Zn, Sr, Cd, Pb, and Cr were also used as non-carcinogen health risk assessment models.

### 2.5. Non-Carcinogenic Risk Assessment

The hazard index (*HI*) is commonly used to assess non-carcinogenic health risks as follows:

(5) $$ADD_{ingestion}=\frac{C_{w}\times IR\times EF\times ED}{BW\times AT},$$

(6) $$ADD_{dermal}=\frac{C_{w}\times SA\times K_{p}\times ET\times EF\times ED\times 10^{-3}}{BW\times AT},$$

(7) $$HQ_{ingestion}=\frac{ADD_{ingestion}}{RfD_{ingestion}};\;HQ_{dermal}=\frac{ADD_{dermal}}{RfD_{dermal}},$$

(8) HI=∑HQ,

The total hazard indexes (*THI*) is calculated by Equation (9)

(9) THI=∑HI,

where *ADD_ingestion_* and *ADD_dermal_* represent the exposure dose for direct ingestion and dermal contact, respectively; *HQ* is the hazard quotient; and *RfD* is the reference dose. The values of the parameters are shown in Table 1, except for those of *K_p_* and *RfD*, which are shown in Table 2 [31,32,33]. For *HI* ≥ 1 or *HQ* ≥ 1, heavy metals posed potential non-carcinogenic risk to humans [1].

### 2.6. Carcinogenic Risk Assessment

The carcinogenic risk level was calculated by:

(10) CR=∑ADD×SF,

The total carcinogenic risk indexes (TCRI) is defined by Equation (11)

(11) TCRI=∑CR, 

where *CR* is the carcinogenic risk and *SF* is the slope coefficient, as shown in Table 2. The acceptable or tolerable carcinogenic risk level was 10^−6^–10^−4^ [34,35].

## 3. Results and Discussion

### 3.1. Distributions of Heavy Metals

The concentrations of heavy metals are shown in Table 3. The average concentrations decreased in the following order: Sr (869.66 µg/dm^3^) > Mn (42.82 µg/dm^3^) > Zn (1.64 µg/dm^3^) > Cu (0.18 µg/dm^3^) > Ni (0.09 µg/dm^3^) > Pb (0.07 µg/dm^3^) > Cr (0.03 µg/dm^3^) > Cd (0.01 µg/dm^3^). The concentrations of Ni, Cu, Zn, Sr, Cd, Pb, and Cr were all below the safety thresholds determined by the World Health Organization [36] and by the Class III thresholds of the national standards for drinking water quality (GB 5749-2006) [37]. In contrast, 23.81% of the samples exceeded the guideline value of 100 μg/dm^3^ for Mn [37], indicating that the shallow groundwater in the study area was slightly polluted by Mn [8]. Sr was identified as the most abundant element, with concentrations >100 μg/dm^3^. Mn and Zn presented a medium abundance, with concentrations of 1–100 µg/dm^3^, whereas Ni, Cu, Cd, Pb, and Cr presented low abundances, with concentrations of <1 µg/dm^3^.

The Ordinary kriging (OK) method uses a semivariogram to consider the spatial distribution of measured values to estimate the unsampled locations values [38,39]. OK uses the position-related mean value and its semivariogram to calculate the best weight and assign the weight to the measurement point, predicting the value of the unknown point [39]. The semivariograms were estimated by the experimental semivariogram calculation *γ*(*h*) as follows:

(12) $$\gamma \left(h\right)=\frac{1}{2n\left(h\right)}\sum_{\left(i,j\right):h_{ij}=h}{\left(x_{i}-x_{j}\right)}^{2}$$

where *γ*(*h*) is the semivariance for distance class *h*, *n*(*h*) is the total number of pairs of values at distance *h*, and *h_ij_* is the distance between locations *i* and *j*.

The accuracy of the model predictions was checked by comparing measured and predicted values determined using the empirical semivariogram model, selecting the model with less error, and then performing cross-validation [14,38].

**Figure 3:**
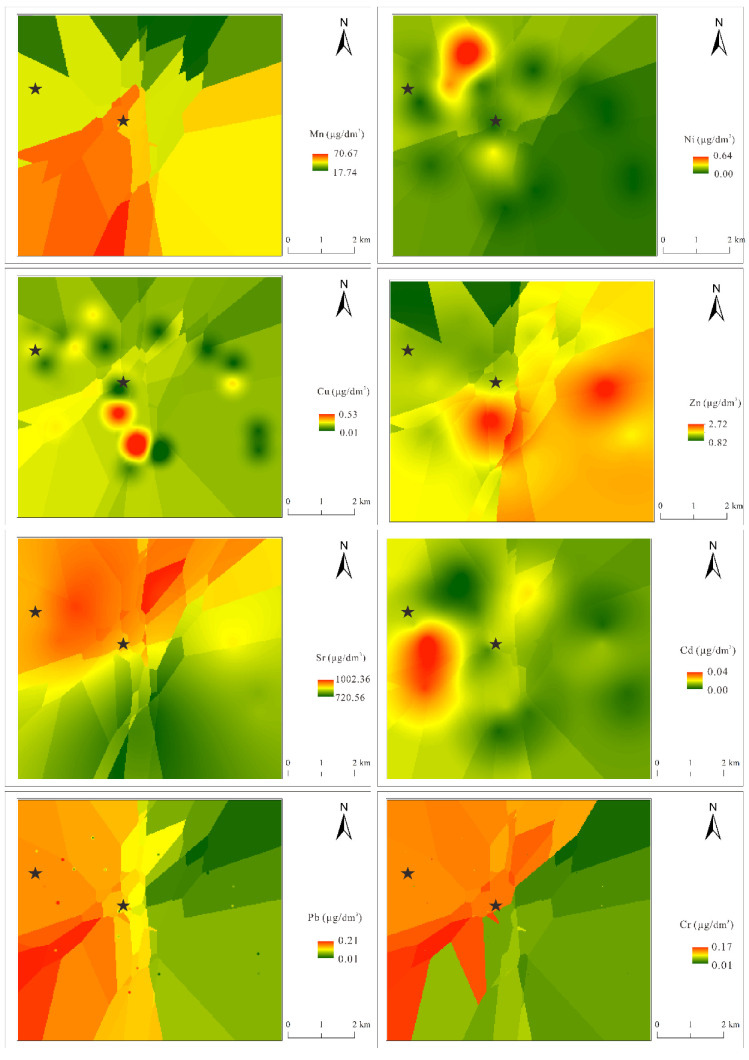
Spatial interpolation distribution of heavy metal concentrations in water samples (the asterisk in the figure represents the location of the poultry farm).

**Figure 4:**
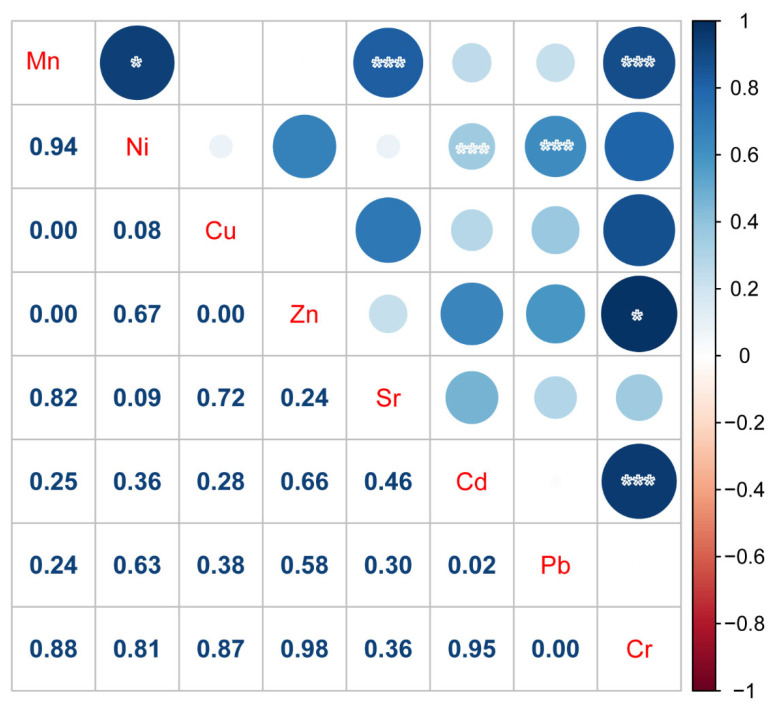
Correlation analysis of eight heavy metals in the poultry farm area. Significant correlation (* *p* ≤ 0.05); (*** *p* ≤ 0.001).

The concentrations of the eight heavy metals were interpolated using the OK method (Figure 3). A normal quantile–quantile (QQ) plot was adopted to test the normal distribution of the dataset, and non-normal datasets were then converted by logarithmic transformation. Figure 3 shows that the spatial distributions of the heavy metals were substantially different. A large area in the southwest (south of the semi-intensive farm) was polluted by Mn. The sewage and wastewater produced by the washing of poultry houses may be directly discharged into the environment, resulting in Mn accumulation in shallow groundwater. Zn pollution was mostly concentrated in the center-right area, as farm sewage water may be unloaded directly into the surface water. The heavy metals were brought into the shallow groundwater through surface water recharge, causing Zn accumulation in the shallow groundwater. Ni pollution was more intense in the northwest, which is close to the coal industrial zone. Studies have shown that industrial activities, such as coal mining, can lead to the accumulation of Ni in the environment [18,40,41]. Therefore, the local high Ni concentration (“hot spot”) was associated with the coal mining industrial activities. Activities such as coal mining also led to the accumulation of heavy metals, such as Pb and Cr [41,42], consistent with the correlation results in Figure 4 [43]. Cr pollution was more severe in the northwest, a densely populated region with tannery and poultry farms. Therefore, Cr pollution likely originated from industrial wastewater and farm sewage. Sr in groundwater exists in the form of lapis lazuli mineral, which is related to the geothermal activity of dissolving limestone, gypsum, and other rocks [44]. The Pb pollution was more intense in the middle-west region of the study area, which was attributed to automobile exhaust (transportation). Most Pb content was accumulated in the southwest area [43].

The literature search yielded a few studies on the heavy metal pollution of shallow groundwater under the impact of poultry farms. Table 4 shows the trace elements in the shallow groundwater of the study area and other similar regions in China and other countries [27,28,29,30,31,32]. The average Mn, Cu, Zn, Pb, and Cr concentrations in this study were much lower than those in the groundwater of polish Western Pomerania [45]. The magnitude of Sr in this study was close to those in groundwater samples of Songyuan City [46]. Furthermore, the Ni, Cu, Cd, Pb, and Cr concentrations measured in this study were lower than those in Guilin, China [47], and India [4], while Zn was slightly higher than in Guilin City.

### 3.2. Source Analysis

The PMF 5.0 model was utilized to identify and assign the sources of eight kinds of shallow groundwater heavy metal pollutants in the study area. The content data for eight heavy metals of 21 samples and the uncertainty data associated were imported into the model. The four-factor solution was determined based on the smallest and most stable Q value, and the signal-to-noise ratios (S/N) of the eight heavy metals were greater than 0.2, which was classified as strong and confirmed the accuracy of the final results. The fitting coefficients of seven heavy metals (R^2^) exceeded 0.70, suggesting a strong correlation among these heavy metals.

**Figure 5:**
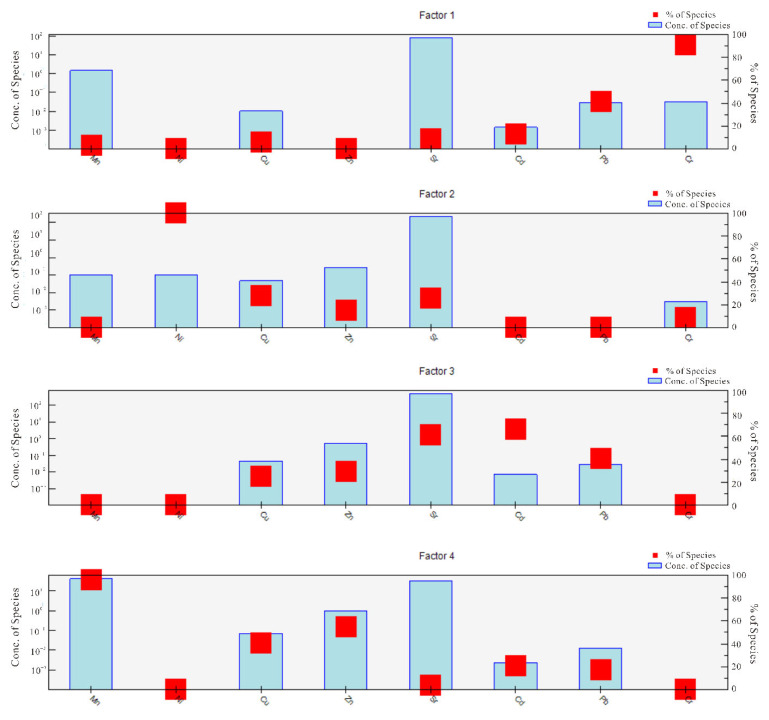
Contribution profile of each factor to heavy metal pollution.

**Figure 6:**
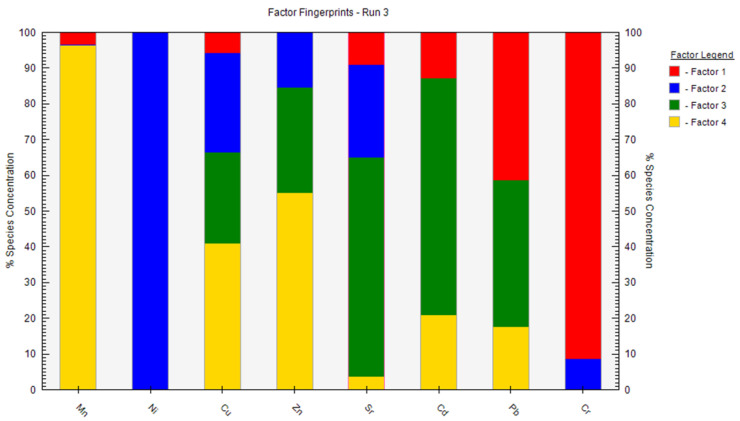
Heavy metal source identification results of PMF model.

We used a PMF model to investigate the sources of heavy metals. As can be seen in Figure 5 and Figure 6, factor 1 is characterized by Cr, and contribution rate of which is 91.3%. As shown in Figure 4, the correlation analysis showed that Cr significantly correlated with Cd, Mn, and Zn (*p* < 0.05). In addition, the spatial distribution of Cd suggested it likely originated from farming activities. Poultry wastewater may be discharged into nearby rivers, lakes, and other water bodies without treatment, resulting in severe heavy metal pollution of surface water. In the wet season, the river diffuses and replenishes the shallow groundwater through infiltration, contaminating the shallow groundwater with its heavy metal load. Therefore, it is speculated that Cr derives from farm sewage. Therefore, the contribution source of Factor 1 was dominated by poultry farms.

The contribution rate of Factor 2 to Ni was 100%. Ni is a common industrial pollutant [43], and the spatial distribution analysis indicated that Ni pollution was concentrated in the northwestern part of the study area, which is a coal mine industrial region, with observed accumulation of coal gangue. The leaching of coal gangue and other coal industrial activities caused the high level of Ni pollution (which exceeded the standard) in the shallow groundwater of this area. Therefore, Factor 2 represented industrial sources.

Factor 3 included significant Cd (66.2%) and Sr (61.3%) loads. Sr mainly comes from natural sources, such as geothermal activity. Therefore, Factor 3 was considered a natural source.

Factor 4 was associated with the high loads of Mn (96.4%) and Zn (55.2%). The Mn- and Zn-contaminated areas were concentrated in the impact zones of the farms. The untreated manure produced by the farms was dumped near the surface water and mixed with the surface water by the action of rainwater runoff, and then brought into the shallow groundwater through surface water replenishment. Therefore, Factor 4 was considered poultry manure [9,25].

Table 5 lists the contribution rate and total contribution rate of each factor to each heavy metal pollution. The most important source of heavy metals in the study area was the waste of poultry farms, accounting for 49.81% of the total, followed by natural sources (27.94%), and industrial activities (22.29%). Therefore, the results indicate that human activities have a considerable impact on the distribution of heavy metals in the shallow groundwater of the study area.

### 3.3. Human Health Risk Assessment

**Figure 7:**
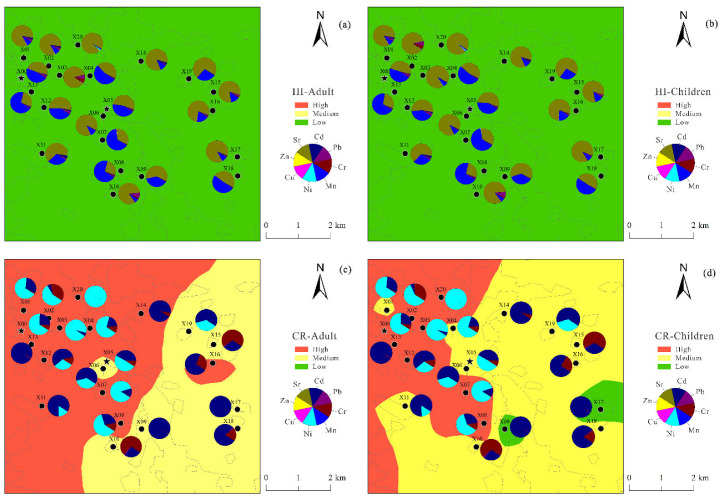
Spatial variation in HI and CR for (**a**,**c**) adults and (**b**,**d**) children.

The contributions of *HQ*, *HI*, *THI*, *CR*, and *TCRI* for adults and children were calculated by considering the contribution of various sources in the PMF model. The two exposure routes (direct drinking and skin contact) were considered, and the obtained values are listed in Table 6. The spatial variations of carcinogenic and non-carcinogenic risks for adults and children are shown in Figure 7.

According to the USEPA, if the *THI* value is less than 1, the non-carcinogenic risk to humans is negligible, whereas values greater than 1 indicate the occurrence of non-carcinogenic risk. The *THI* values for adults and children were 5.59 × 10^−1^ and 7.97 × 10^−1^, respectively, which are lower than 1. Therefore, the eight heavy metals in the shallow groundwater of the farm area did not pose a significant non-carcinogenic risk to the human body. However, children had higher *THI* values than adults (close to 1); thus, the non-carcinogenic risk to children from shallow groundwater in the study area cannot be ignored [43]. The values of *CR* for adults and children were 1.68 × 10^−2^ and 4.93 × 10^−3^, respectively, which exceeded the acceptable risk levels. Therefore, the shallow groundwater of the study area presented carcinogenic risks to adults and children. Adults had higher *TCRI* values than children, and the carcinogenic risk for adults was relatively higher. For non-carcinogenic risks, the *HQ* values of the four factors were significantly higher for children than for adults, whereas for carcinogenic risks, the contribution values of the four factors were significantly higher for adults than for children.

**Figure 8:**
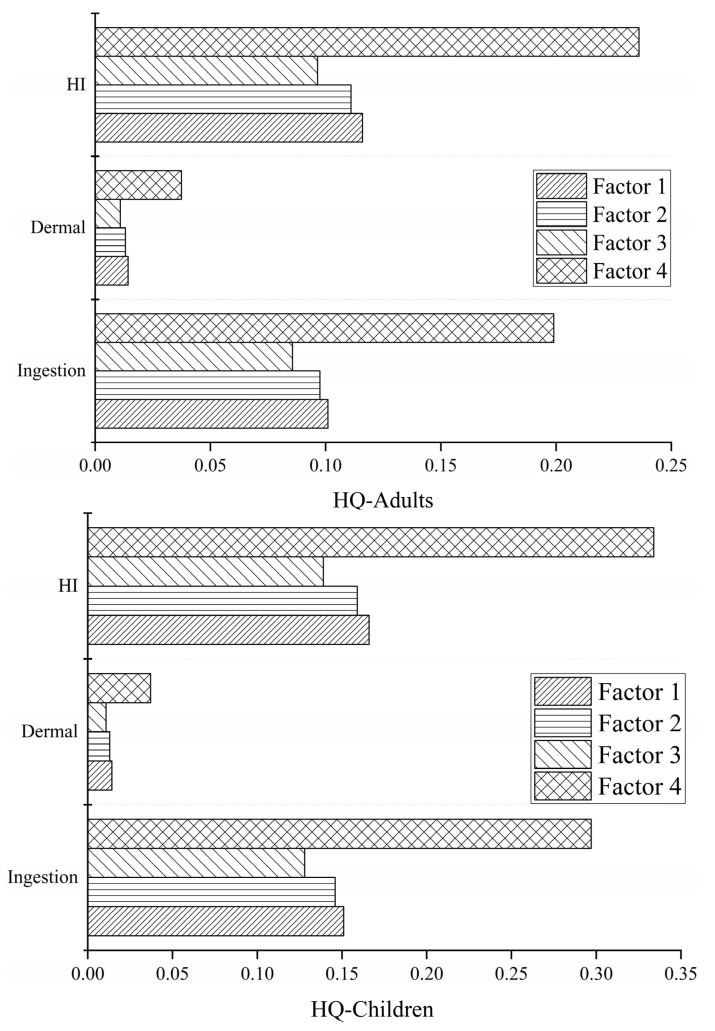
*HI* and *HQ* values for adults and children.

**Figure 9:**
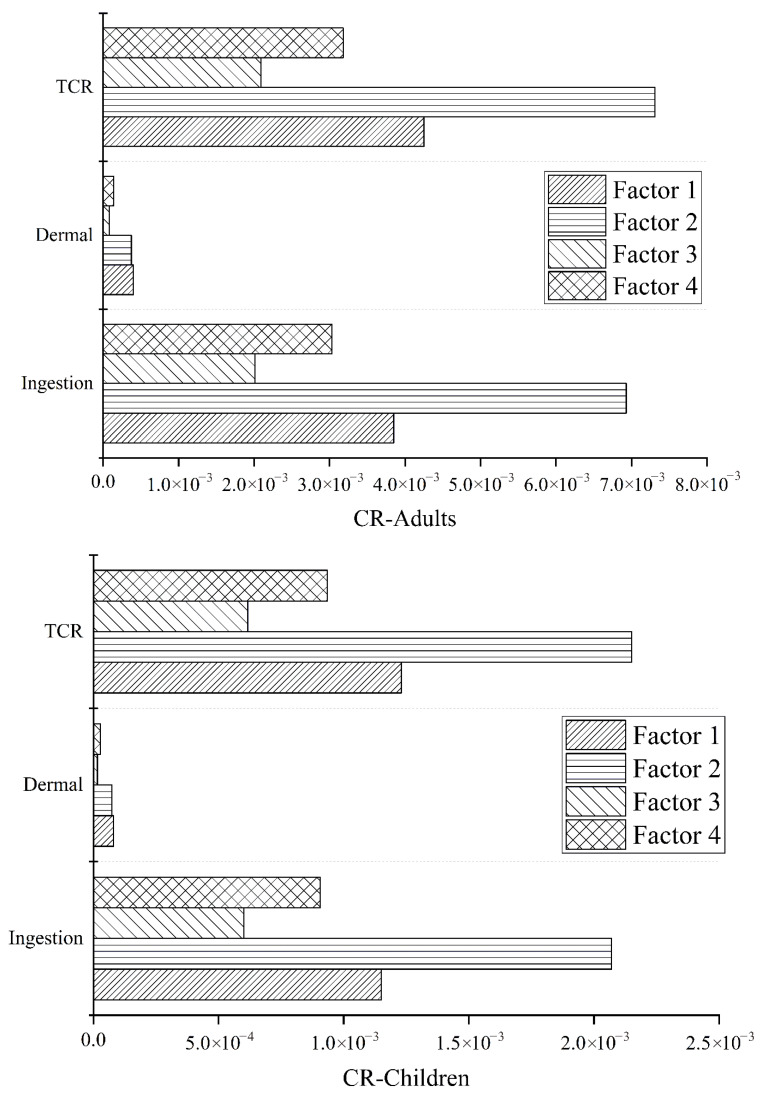
*HI* and *CR* values for adults and children.

Figure 8 and Figure 9 show that for *HQ*, the non-carcinogenic risk of adults and children via drinking water was higher than that via skin contact. The non-carcinogenic risk of the four factors via drinking water was greater for children than for adults, and the opposite occurred for the non-carcinogenic risk via skin contact. The carcinogenic risks of the four factors via direct ingestion and skin contact were greater for adults than for children. In addition, the contribution of direct ingestion to adult *HQ* and *CR* was the largest, accounting for 86% and 94% of the total, respectively. The same was observed for children, with contributions of 91% and 96%, respectively.

### 3.4. Health Risk Assessment of Heavy Metals from Different Sources

**Figure 10:**
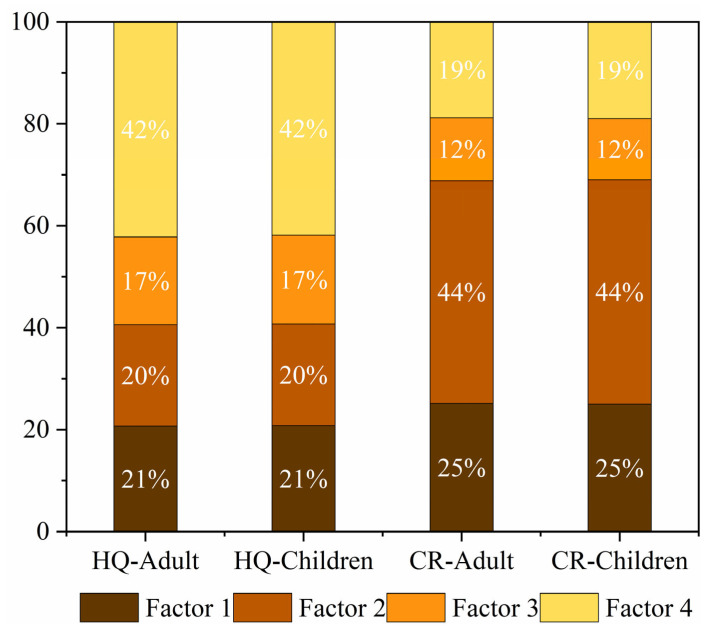
Contribution ratio of heavy metal sources to HI and CR values for adults and children.

Four different sources of heavy metals were identified using the PMF model, and the non-carcinogenic (according to the *HQ* value) and carcinogenic (according to the *CR* value) risk contributions of these four sources to adults and children were calculated (Table 6, Figure 7 and Figure 8). Factor 4 contributed the most to the overall non-carcinogenic risk, accounting for 42% of the total, followed by Factor 1 (21%). Factors 1 and 2 contributed significantly to the overall carcinogenic risk, accounting for 25% and 44%, respectively (Figure 10).

The results show that poultry farming was the main source of heavy metals in the region, and it was the main factor causing non-carcinogenic risks to adults and children. The most important factor posing carcinogenic risks to adults and children was industrial activities. Therefore, local authorities should strengthen measures for industrial environmental protection.

### 3.5. Uncertainty Analysis

The health risks of heavy metals in shallow groundwater in the area affected by poultry farms were analyzed in this study. First, uncertainty lies in the accuracy and precision of the instrument (such as the LOQ value). Second, the uncertainty of the results mainly originates from the analysis method because the analyzed risks included only drinking and skin contact. Therefore, other exposure routes were excluded, including intake or inhalation of soil, air, food, and other media. In addition, the dietary habits of residents were not fully considered. For instance, the concentration of some pollutants can be reduced if the groundwater is boiled before ingestion, and the absorption factors of the human digestive system can affect the uptake of other pollutants. Therefore, future studies should include more data and habits so as to provide a more accurate risk assessment.

## 4. Conclusions

The quality of shallow groundwater can directly affect the health of surrounding communities. In this study, we investigated the sources of heavy metal pollution to shallow groundwater under the impact of poultry farms and the respective health risks. The Mn content exceeded the standard limit in 23.81% of the samples, whereas the concentrations of the other elements did not exceed the standard. OK interpolation was used to analyze the concentration distribution of eight heavy metals, and the results showed that human activities substantially contributed to the pollution of groundwater. The non-carcinogenic risks to adults and children were lower than the risk thresholds, whereas the carcinogenic risks exceeded the limit. Therefore, the heavy metal pollution in the study area presented a carcinogenic risk. The PMF results showed that poultry manure was the largest contributor to heavy metal pollution in shallow groundwater in the impact zone of poultry farms in Zhuxianzhuang, accounting for 29.33% of the total heavy metal load, followed by natural sources (27.94%), industrial sources (22.29%), and poultry wastewater (20.48%). The health risk assessment revealed that children had a higher non-carcinogenic risk than adults, whereas adults faced a higher risk of cancer than children. Poultry farms presented the greatest contribution to non-carcinogenic risks (63%), whereas industrial activity (44%) was the primary contributor to carcinogenic risks. Consequently, poultry farms and industrial activities should be the primary targets of pollution control and risk prevention.

## Figures and Tables

**Table 1:** Distributions of parameters for the health risk assessment.

Symbol	Parameter	Units	Distribution	
			Adult	Child
C_w_	average concentration	μg/L		
IR	intake rate	L/day	2	0.64
EF	exposure frequency	days/year	350	350
ED	duration of exposure	years	30	6
SA	exposed area of skin	cm^2^	18,000	6600
ET	exposure time	h/day	1	0.58
BW	body weight	kg	70	15
AT (noncarcinogenic)	average time	days	10,950	2190
AT (carcinogenic)	average time	days	25,550	25,550

**Table 2:** Standard values of RfD, Kp, and SF in the study.

	RfD_ingestion_ (μg/kg/day)	RfD_dermal_ (μg/kg/day)	Kp ^d^ (cm/h)	SF_ingestion_ (mg/kg/d)^−1^	SF_dermal_ (mg/kg/d)^−1^
Mn ^a^	24	0.96	1 × 10^−3^		
Ni ^a^	20	0.8	2 × 10^−4^	1.7	42.5
Cu ^a^	40	8	1 × 10^−3^		
Zn ^a^	300	60	6 × 10^−4^		
Sr ^b,c^	600	120	1 × 10^−3^		
Cd ^a^	0.5	0.025	1 × 10^−3^	6.1	0.38
Pb ^a^	1.4	0.42	1 × 10^−4^	8.5 × 10^−3^	0.073
Cr ^a^	3	0.075	1 × 10^−3^	0.5	20

^a^ [17]; ^b^ [31]; ^c^ [32]; ^d^ [33].

**Table 3:** Statistical analysis of heavy metal content in shallow groundwater in coal–poultry farming districts.

Heavy Metals	Range (µg/dm^3^)	SD	Class ^a^	WHO ^b^
Mn	0.03–234.19	69.73	100	500
Ni	0.00–0.65	0.16	20	20
Cu	0.01–0.55	0.12	1000	1000
Zn	0.29–7.26	1.87	1000	5000
Sr	360.09–1528.99	323.21	-	-
Cd	0.00–0.04	0.01	5	5
Pb	0.01–0.25	0.06	10	10
Cr	0.00–0.31	0.07	50	50

^a^ [37]; ^b^ [36].

**Table 4:** Heavy metal concentrations in shallow groundwater in previous studies carried out in world areas.

Location	Mn	Ni	Cu	Zn	Sr	Cd	Pb	Cr	References
Western Pomerania	160	-	10	57	224	-	37	5	[45]
Songyuan City	-	-	-	-	900	-	-	-	[46]
Guilin City	-	-	0.36	0.63	-	0.06	0.12	-	[47]
India	-	5.71	26.85	124.25	-	0.067	14.36	>10	[4]
This study	42.82	0.09	0.18	1.64	869.66	0.01	0.07	0.03	This study

Units: μg/dm^3^.

**Table 5:** Source contribution ratios (%) of heavy metals.

Source	Mn	Ni	Cu	Zn	Sr	Cd	Pb	Cr	Contribution Ratios
Factor 1	3.30	0	5.80	0	9.00	13.00	41.40	91.30	20.48
Factor 2	0.20	100	27.80	15.50	26.10	0	0	8.70	22.29
Factor 3	0	0	25.50	29.40	61.30	66.20	41.10	0	27.94
Factor 4	96.40	0	41.00	55.20	3.60	20.80	17.60	0	29.33

**Table 6:** *HQ*, *HI*, *THI*, *CR*, and *TCRI* for adults and children calculated by PMF mode.

	Adult			Children		
Pathway	Ingestion	Dermal	Total	Ingestion	Dermal	Total
Noncarcinogenic risk (*HQ*) ^a^						
Factor 1	1.01 × 10^−1^	1.43 × 10^−2^	1.16 × 10^−1^	1.51 × 10^−1^	1.42 × 10^−2^	1.66 × 10^−1^
Factor 2	9.75 × 10^−2^	1.31 × 10^−2^	1.11 × 10^−1^	1.46 × 10^−1^	1.30 × 10^−2^	1.59 × 10^−1^
Factor 3	8.56 × 10^−2^	1.09 × 10^−2^	9.65 × 10^−2^	1.28 × 10^−1^	1.08 × 10^−2^	1.39 × 10^−1^
Factor 4	1.99 × 10^−1^	3.74 × 10^−2^	2.36 × 10^−1^	2.97 × 10^−1^	3.71 × 10^−2^	3.34 × 10^−1^
THI	4.83 × 10^−1^	7.57 × 10^−2^	5.59 × 10^−1^	7.22 × 10^−1^	7.51 × 10^−2^	7.97 × 10^−1^
Carcinogenic risk (*CR*) ^a^						
Factor 1	3.85 × 10^−3^	4.03 × 10^−4^	4.25 × 10^−3^	1.15 × 10^−3^	8.03 × 10^−5^	1.23 × 10^−3^
Factor 2	6.93 × 10^−3^	3.75 × 10^−4^	7.31 × 10^−3^	2.07 × 10^−3^	7.46 × 10^−5^	2.15 × 10^−3^
Factor 3	2.01 × 10^−3^	8.16 × 10^−5^	2.09 × 10^−3^	6.01 × 10^−4^	1.63 × 10^−5^	6.17 × 10^−4^
Factor 4	3.03 × 10^−3^	1.41 × 10^−4^	3.18 × 10^−3^	9.06 × 10^−4^	2.81 × 10^−5^	9.35 × 10^−4^
TCRI	1.58 × 10^−2^	1.00 × 10^−3^	1.68 × 10^−2^	4.73 × 10^−3^	1.99 × 10^−4^	4.93 × 10^−3^

^a^ means *HQ*, *CR* were average value of water samples.

## Data Availability

The datasets used and/or analyzed in this study are available from the corresponding author on reasonable request.
